# Orthogonal Study on Pore Structure and Compressive Strength of Densifier Modified Fly Ash Concrete

**DOI:** 10.3390/ma19214523

**Published:** 2026-10-27

**Authors:** Benquan Xu, Yusong Miao, Chong Gao, Chunbo Zhou, Jian Guo, Chaoyue Xie

**Affiliations:** 1 School of Science, Qingdao University of Technology, Qingdao 266525, China; 15689712179@163.com (B.X.); gaochonggc_2008@163.com (C.G.); a909312365@163.com (C.X.); 2 China Railway 19th Bureau Group Corporation Limited, Beijing 100192, China; 3 Institute of Blasting Technology, Liaoning Technical University, Fuxin 123000, China; gj_hsa@163.com

**Keywords:** fly ash, orthogonal experimental design, pore structure, X-ray computed tomography (X-CT), densifier

## Abstract

The effects of MATE-ADV densifier dosage on CT-resolvable pore characteristics and compressive strength in fly ash–slag concrete remain insufficiently quantified. An L9 orthogonal array was used to evaluate cement content, sand ratio, fly ash (FA) content, and densifier dosage. X-ray computed tomography (X-CT) characterized the specimens at 28 d, and compressive strength was measured at 7, 14, and 28 d. Across the nine specimen groups, mean slice porosity ranged from 0.75% to 2.50%, while 28 d compressive strength ranged from 59.49 to 68.37 MPa. Exploratory pooled-error ANOVA suggested main effects of cement content (F_(2,4)_ = 8.05, *p* = 0.040) and sand ratio (F_(2,4)_ = 7.71, *p* = 0.042) on mean slice porosity. Cement content and sand ratio showed the largest main effects on 28 d strength; FA content was also significant, whereas densifier dosage was not (F_(2,18)_ = 2.79, *p* = 0.088). X-CT analysis showed variation in pore-section area and circularity among the groups. A second-order logarithmic model summarized the observed porosity–strength relationship (R^2^ = 0.948; leave-one-group-out RMSE = 1.422 MPa). Finally, a candidate for the optimal mix proportion was identified by jointly considering the evaluated 2D pore characteristics and 28 d compressive strength.

## 1. Introduction

The mechanical performance of concrete depends on its constituent proportions and the pore system formed in the hardened material [1,2]. Classical strength–porosity relationships describe the broad influence of void content on strength [3], whereas pore size and shape provide further information about mechanical behavior [4]. Binder composition and functional admixtures warrant product-specific evaluation. Studies have examined fly ash in slag-cement concrete [5], the 28-day strength of concrete with a crystalline admixture [6], and both pore refinement and strength in nano-TiO_2_-modified concrete [7]. X-ray computed tomography (X-CT) offers a non-destructive way to examine voids detectable at the chosen resolution [8]. Prior work has compared two- and three-dimensional air-void descriptors [9] and combined orthogonal mixture design with X-CT analysis in other cement-based systems [10]. Together, these studies motivate examining how densifier dosage and mix proportions relate to CT-resolvable void characteristics and compressive strength in fly ash–slag concrete within a single orthogonal experiment.

Key factors such as cement content, sand ratio, and fly ash (FA) dosage can affect concrete pore characteristics and compressive strength [11,12], while MATE-ADV densifier dosage is examined as an additional factor in the present study. Changes in cement content can alter the paste proportion and compressive strength [11]. Studies of ternary cementitious systems have also related particle packing to CT-measured pore characteristics [13]. The water-to-binder ratio can also affect pore development in fly ash concrete, as shown by industrial CT observations [14]. Furthermore, the gradation and volume fraction of sand are pivotal to the internal skeleton structure. Wang et al. [15] investigated the sand-to-aggregate ratio in concrete and found that the ratio corresponding to the highest compressive strength varied with water content. Similarly, Ye et al. [16] employed fractal analysis to examine alkali-activated slag cementitious materials and reported that fine sand reduced total porosity and the proportion of large pores. Although recent studies have examined mixture variables affecting compressive strength [11,15] and fly-ash-related pore characteristics [12], these four factors have not been compared together for the FA–GGBS concrete and MATE-ADV dosage range investigated here. Therefore, an orthogonal experimental design was adopted in the present study to screen these four factors efficiently and compare their main-effect trends in relation to the 2D pore characteristics and compressive strength.

FA content can affect pore characteristics and compressive strength, with the observed response depending on the concrete composition and dosage [12,17]. Guo et al. [17] found that using a combination of FA, slag, and silica fume (SF) compensated for the adverse effects of recycled concrete aggregates (RCAs) and improved the mechanical properties of recycled aggregate self-compacting concrete (RA-SCC). Kang et al. [18] investigated high-performance concrete with different FA contents and reported corresponding changes in compressive strength and pore-size distribution using low-field nuclear magnetic resonance and SEM. Previous studies have shown that fly ash particle size and binder design can modify pore characteristics in cementitious matrices, with corresponding changes in compressive strength [19,20]. However, the respective main-effect trends of fly ash and commercial densifier dosage on CT-derived pore characteristics and compressive strength remain insufficiently quantified.

Previous studies have reported an association between porosity, pore size, and compressive performance in concrete [21]. X-ray computed tomography (X-CT) enables the non-destructive characterization of void size and spatial distribution in concrete at the selected scan resolution [22]. Huo et al. [23] integrated three-dimensional reconstruction, fractal analysis, and multi-parameter regression modeling to quantify the correlation between pore parameters and material properties. Two-dimensional image processing can quantify pore area fraction and pore size on cross-sections [24]. In the present study, area and circularity are measured from the X-CT slices. Rong et al. [4] investigated the effects of sand-to-binder ratio and water-to-binder ratio on the workability and mechanical properties of ultra-high performance cementitious composites (UHPCCs) based on the modified Andreasen and Andersen grading model (MAA) and orthogonal experimental design, and quantitatively analyzed steel fiber distribution and mesoscopic pore structure using X-CT technology. The present study applies an L9 orthogonal design and X-CT slice analysis to FA–GGBS concrete containing MATE-ADV densifier, comparing the main-effect trends of cement content, sand ratio, FA content, and densifier dosage in relation to 2D pore descriptors and compressive strength.

This study uses an L9 orthogonal design, range analysis, and analysis of variance (ANOVA) to compare the main-effect trends of cement content, sand ratio, FA content, and MATE-ADV densifier dosage. X-CT images acquired at 28 d are processed to quantify slice-wise 2D porosity, pore-section area, and circularity. Compressive strength is measured at 7, 14, and 28 d, and its 28 d results are compared with the CT-derived pore descriptors. In particular, the study tests whether MATE-ADV dosage has a detectable effect on pore characteristics and compressive strength within the investigated range. We hypothesized that the four factors would show different main-effect trends and that average 2D porosity would be associated with 28 d compressive strength.

## 2. Specimen Preparation and Experimental Program

### 2.1. Materials and Specimen Preparation

The raw materials used in this study include cement, river sand, coarse aggregate, water, FA, ground granulated blast-furnace slag (GGBS), superplasticizer, and MATE-ADV densifier. The physicochemical properties of these materials are summarized in Table 1. Concrete specimens were prepared according to a sequential dry-mixing followed by wet-mixing procedure. Initially, the solid constituents, including cement, sand, coarse aggregate, FA, and GGBS, were homogenized in a concrete mixer to ensure thorough contact between aggregates and cementitious materials. Subsequently, the superplasticizer and densifier were pre-dissolved in the mixing water and incrementally added to the mixer. The mixture was continuously mixed until satisfactory workability was achieved. Fresh concrete was cast into 50 mm × 50 mm × 50 mm non-standard cubic molds. Afterwards, the specimens were vibrated on a high-frequency vibration table for 25 s to remove entrapped air and ensure structural density. After 24 h, specimens were demolded and cured in a standard curing room (20 ± 2 °C, RH > 95%) until the designated testing ages (7, 14, and 28 days).

### 2.2. Mix Proportion Design and Experimental Methodology

#### 2.2.1. Orthogonal Experimental Design

The compressive strength and CT-derived pore characteristics of concrete may be influenced by cement content, sand ratio, fly ash dosage, and densifier dosage [25,26,27]. However, full-factorial experimentation is often prohibitive due to the exponential increase in required tests. The influences of these four factors on compressive strength and porosity were efficiently evaluated using an orthogonal experimental design. The combination of factor levels was determined through nine groups of tests by selecting an L9 (3^4^) orthogonal array. This approach significantly reduces experimental costs while enabling the isolation of main-effect contributions via subsequent statistical analysis. Consequently, the design provides a quantitative basis for screening candidate mix proportions within the tested factor ranges. The sand ratio was defined on a mass basis as the proportion of fine aggregate in the total aggregate mass:

(1) $$\beta_{s}=\frac{m_{s}}{m_{s}+m_{g}}\times 100\%$$

where $\beta_{s}$ is the sand ratio, and $m_{s}$ and $m_{g}$ are the masses of sand and coarse aggregate, respectively. Because the coarse-aggregate mass was fixed at 975 g per laboratory batch, the sand mass was calculated from the specified sand ratio as

(2) $$m_{s}=\frac{\beta_{s}}{100-\beta_{s}}m_{g}$$

Accordingly, sand ratios of 43.51%, 42.25%, and 40.94% corresponded to sand masses of approximately 751.0, 713.45, and 675.9 g per batch, respectively.

Three independently mixed batches were prepared for each of the nine mixture combinations. At each testing age, one cube from each batch was tested, providing three independent compressive-strength measurements for each mixture and age. All 27 individual 28 d cube strengths (three independently mixed batches for each of nine mixtures) were used for the strength ANOVA, yielding 18 within-mixture error degrees of freedom; the plotted factor-level trends summarize the balanced mixture means. The detailed experimental design matrix is presented in Table 2. All quantities reported in Table 2 represent the actual masses used in one laboratory batch. The total binder content was defined as the combined mass of Portland cement, fly ash, and ground granulated blast-furnace slag:

(3) $$m_{b}=m_{c}+m_{\mathrm{FA}}+m_{\mathrm{GGBS}}$$

The reported nominal water-to-binder ratio of 0.37 was calculated using the separately added tap-water mass. The carrier water contained in the liquid superplasticizer and densifier was not deducted from the mixing water. The exact effective water-to-binder ratio could not be calculated because the carrier-water fractions of the liquid admixtures were unavailable. Even if the entire maximum densifier mass (1.8 g) were counted as water, its contribution would be below 0.0044 relative to the minimum binder mass (409.5 g); the superplasticizer dosage was constant across mixtures. Any carrier-water effect remains inseparable from the densifier-dose comparison.

The factor-level codes used throughout the manuscript were defined according to the order presented in Table 2. Specifically, A_1_, A_2_, and A_3_ corresponded to cement contents of 337, 320.15, and 303.3 g, respectively; B_1_, B_2_, and B_3_ corresponded to sand ratios of 43.51%, 42.25%, and 40.94%; C_1_, C_2_, and C_3_ corresponded to FA contents of 58.0, 55.1, and 52.2 g; and D_1_, D_2_, and D_3_ corresponded to densifier dosages of 0, 0.9, and 1.8 g. The FA and densifier levels in Table 2 denote the masses of the supplied materials per laboratory batch, not percentages of binder or cement replacement.

#### 2.2.2. Range Analysis

Range analysis was used to compare the main-effect trends of the four factors within the nine-run design. The factor-level responses provide a concise comparison of variation in compressive strength and CT-derived pore characteristics. The primary controlling factors affecting concrete performance can be determined, and response-specific candidate factor levels can be identified, through the calculation of the average response values ($K_{ji}$) and the range ($R_{j}$) for each factor at different levels; the computational procedure is as follows: first, the mean value of the experimental results for each factor at each level is calculated, denoted as $\bar{K_{ji}}$.

(4) $$R_{j}=\max\left\{\bar{K_{ji}}\right\}-\min\left\{\bar{K_{ji}}\right\}$$

where $R_{j}$ denotes the range of factor j; $\max\left\{\bar{K_{ji}}\right\}$ and $\min\left\{\bar{K_{ji}}\right\}$ represent the maximum and minimum mean response values for factor j at level i, respectively; j=A,B,C,D; and i=1,2,3. The factors were ranked by comparing their range values.

#### 2.2.3. Analysis of Variance

Range analysis successfully provided the hierarchical order of influence, but it cannot distinguish whether the observed variances are derived from the deterministic effects of factor variations or the stochastic nature of experimental error. Analysis of variance (ANOVA) [28] was used to assess the statistical significance of each mix proportion parameter. The total variance is partitioned into factor main effects and random error components, alongside the construction of the *F*-statistic for hypothesis testing (*F*-test).

The *F*-statistic is defined as follows:

(5) $$F_{j}=\frac{V_{j}}{V_{e}}$$

where $V_{j}$ denotes the mean square of factor j, and $V_{e}$ represents the mean square error. If the calculated *F*-value exceeds the critical value $F_{\alpha }\left(df_{j},df_{e}\right)$, the corresponding factor is deemed to have a statistically significant effect on the experimental results.

For each factor, the experimental results are grouped according to the respective levels, and the sum of the results for each level is subsequently calculated. The sum of squares (SS) for each factor is given by

(6) $$SS_{j}=\sum_{i=1}^{3}\frac{T_{ji}^{2}}{n_{j}}-\frac{T^{2}}{N}$$

where $T_{ji}$ is the sum of the observed responses at level i of factor j, $n_{j}$ is the number of observations at each level, T is the sum of all observations, and N is the total number of observations. For the 28 d compressive-strength analysis, each factor level contains nine batch-level observations; therefore, $n_{j}=9$ and N=27.

The sum of squares for error ($SS_{e}$) is expressed as:

(7) $$SS_{e}=\sum_{g=1}^{9}\sum_{b=1}^{3}{\left(f_{c,gb}-{\bar{f}}_{c,g}\right)}^{2}$$

where $f_{c,gb}$ denotes the measured 28 d compressive strength of the cube from independently mixed batch b of mixture g, and ${\bar{f}}_{c,g}$ denotes the mean of the three batch-level strengths for that mixture. Here, g=1,…,9 and b = 1,2,3; thus, the error term has 9(3–1) = 18 degrees of freedom.

The mean square (V) for each factor and the corresponding error can be expressed as:

(8) $$V_{j}=\frac{SS_{j}}{df_{j}}=\frac{SS_{j}}{2}$$

(9) $$V_{e}=\frac{SS_{e}}{df_{e}}$$

where $df_{j}(df_{j}=r-1)$ and $df_{e}$ ($df_{e}=df_{T}-\sum df_{j}$) denote the degrees of freedom for each factor and the error term, respectively.

Because the factor-level combinations predicted for the different responses were not identical, a qualitative consistency analysis was used to identify a multi-response candidate combination. For each factor, the frequency with which each level appeared in the four response-specific candidate combinations was compared. When no single level occurred most frequently, the level was selected according to the primary objective of pore-structure refinement, while also considering the statistical significance of its effect on compressive strength.

#### 2.2.4. Experimental Procedure

**Figure 1:**
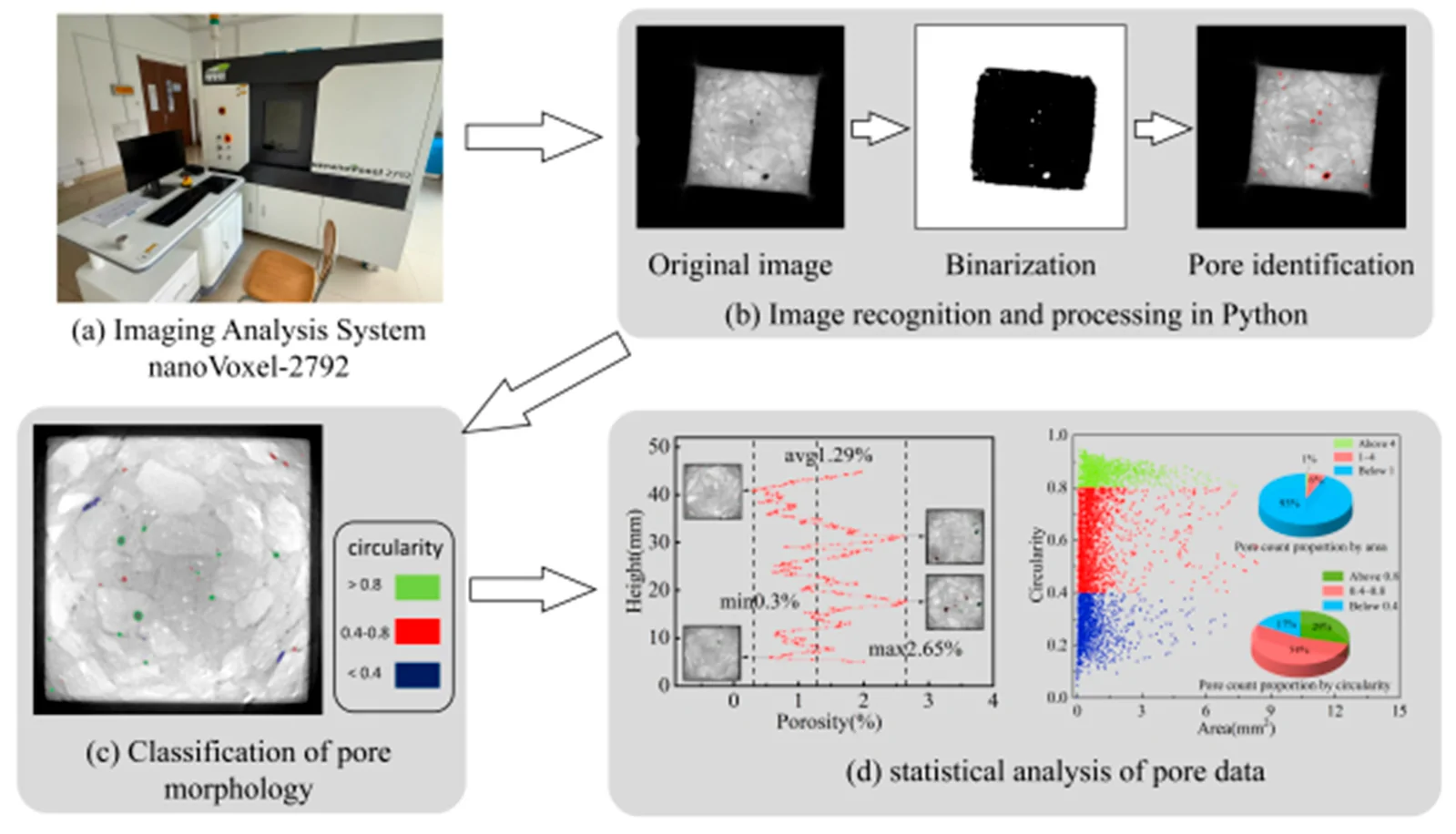
Workflow for CT-based two-dimensional pore-section analysis: (**a**) CT acquisition and reconstruction; (**b**) grayscale filtering and histogram equalization; (**c**) threshold segmentation and specimen masking; and (**d**) extraction of pore-section area and circularity.

X-ray computed tomography was performed at 28 d before the compression tests using a nanoVoxel-2792 imaging system (Figure 1a). The tube voltage and current were 130 kV and 100 μA, respectively, and the exposure time was 0.50 s per projection. A total of 1080 projections were acquired. The manufacturer-specified spatial resolution of 3 μm represents the maximum capability of the instrument rather than the voxel size used in the present scans. The reconstructed volumes consisted of 768 × 768 × 768 isotropic voxels with an actual voxel dimension of 0.11043 mm. One independently prepared specimen from each mixture was randomly selected for CT scanning.

The reconstructed axial slices were processed using a custom Python (3.12.7) workflow based on OpenCV. For each specimen, 562 slices corresponding to image indices 88–649 were retained for analysis. The slices were imported as 8-bit grayscale TIFF images, denoised using a 5 × 5 Gaussian filter, and subjected to slice-wise histogram equalization. The same fixed threshold of 126 was applied to the equalized grayscale images of all nine specimens. The binary images were subsequently inverted and processed using a 3 × 3 morphological closing operation. The specimen cross-sectional mask was used to exclude the surrounding air from the analysis (Figure 1b).

Connected components containing fewer than 8 pixels were excluded as resolution-related noise. Based on the in-plane pixel size of 0.11043 mm, this criterion corresponds to a minimum equivalent pore-section diameter of approximately 0.35 mm. Because circularity is more sensitive to boundary discretization, circularity was evaluated only for components containing at least 29 pixels, corresponding to an equivalent diameter of approximately 0.67 mm.

For each retained slice, the two-dimensional porosity was calculated as

(10) $$\phi_{k}=\frac{A_{\mathrm{pore},k}}{A_{\mathrm{specimen},k}}\times 100\%$$

where $A_{\mathrm{pore},k}$ is the total segmented pore area and $A_{\mathrm{specimen},k}$ is the specimen-mask area on slice (*k*). The average slice porosity of each specimen was obtained by arithmetically averaging the porosities of all 562 retained slices.

The physical area of each pore section was calculated as $A=N_{p}s^{2}$, where $N_{p}$ is the number of pixels belonging to the pore section and (s = 0.11043) mm is the in-plane pixel dimension. Connected components containing fewer than 8 pixels, corresponding to a pore-section area of approximately 0.10 mm^2^, were excluded as resolution-related noise. Because circularity is more sensitive to boundary discretization, circularity was evaluated only for pore sections containing at least 29 pixels, corresponding to an area of approximately 0.354 mm^2^.

For comparative analysis, the retained pore sections were classified into three operational area intervals: A<1 mm^2^, 1≤A<4 mm^2^, and A≥4 mm^2^. These class limits were applied consistently to all mixtures to distinguish relatively small, intermediate, and large CT-resolvable pore sections.

Pore-section circularity was calculated as

(11) $$C=\frac{4\pi A}{P^{2}}$$

where P is the perimeter of the pore section. The pore sections were classified as highly irregular (C<0.4), moderately irregular (0.4≤C<0.8), and near-circular (C≥0.8). The thresholds of 0.4 and 0.8 were used as operational shape classes for comparing the mixtures. These classes describe two-dimensional section geometry and do not independently identify cracks, microcracks, or interfacial-transition-zone defects.

The reported counts represent two-dimensional pore sections identified independently on individual CT slices rather than unique three-dimensional pores. A three-dimensional void may therefore contribute sections to several adjacent slices. This does not affect the calculation of slice porosity because the pore-area fraction was independently calculated for every slice, but the reported counts should not be interpreted as the number of unique three-dimensional pores. The slices were treated as spatial observations within one specimen rather than independent specimen-level replicates.

**Figure 2:**
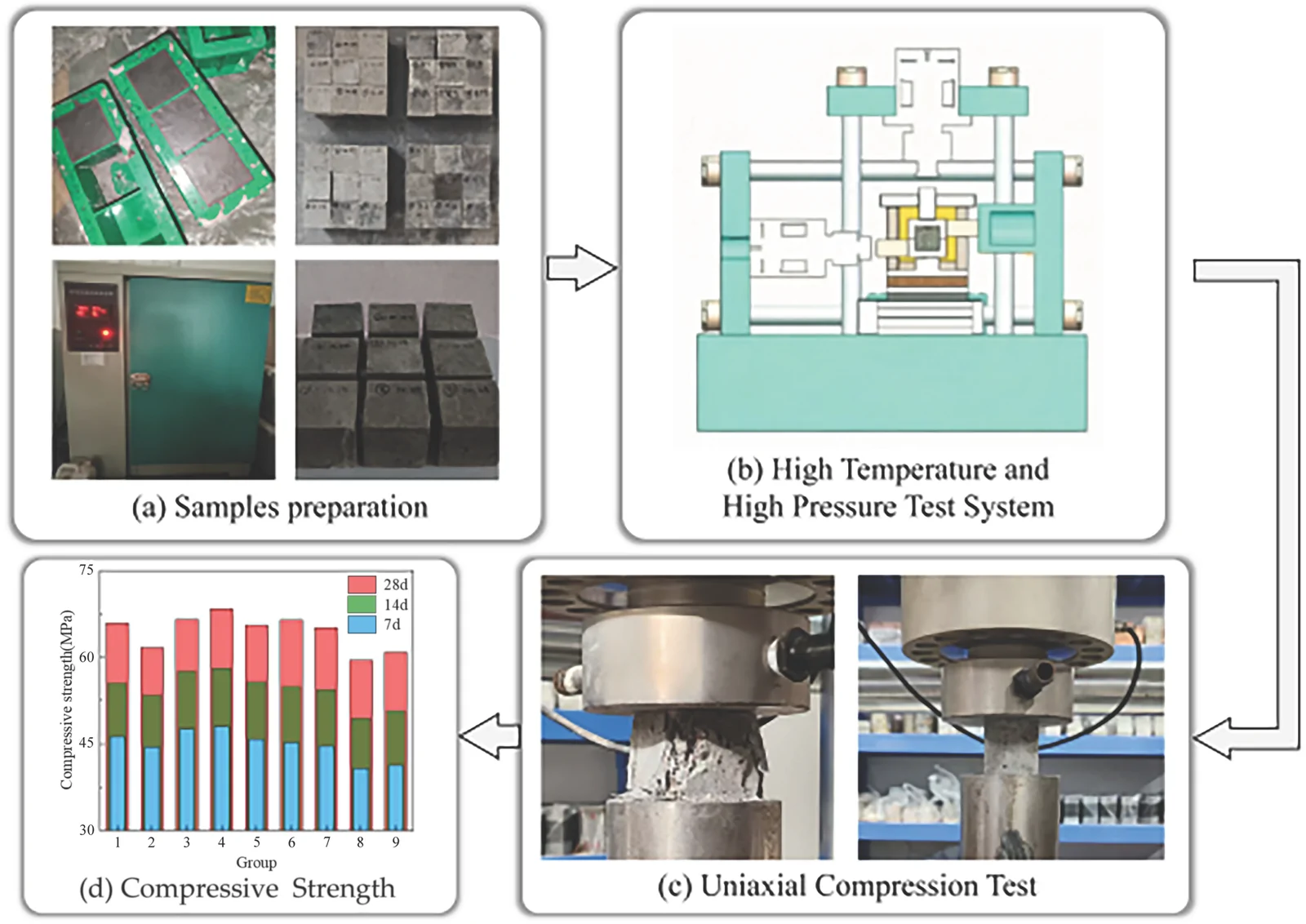
The experimental procedure for uniaxial compressive strength testing: (**a**) preparation and curing of concrete specimens; (**b**) schematic of the high-temperature and high-pressure testing system; (**c**) uniaxial compression testing of concrete specimens; (**d**) compressive strengths of the nine specimen groups at curing ages of 7, 14, and 28 days.

The procedure for the uniaxial compressive strength (UCS) test is illustrated in Figure 2. Tests were performed utilizing a custom-built high-temperature and high-pressure testing system (Figure 2b). The system supports displacement loading rates of 0.001–1 mm/s and force loading rates of 0.01–10.0 kN/s, with a control accuracy of 0.5% of its maximum load capacity. Tests were conducted under displacement control at a constant loading rate of 0.003 mm/s, with reference to the Standard for Test Methods of Concrete Physical and Mechanical Properties [29]. Prior to testing, the effective load-bearing surface dimensions were precisely measured using Vernier calipers. To eliminate discrepancies induced by dimensional variations, the actual cross-sectional area of each specimen was applied in subsequent strength calculations. For each experimental group, three specimens were subjected to the UCS test (Figure 2c), and the average value was taken as the representative compressive strength, calculated as follows:

(12) $$f_{c}=\frac{F}{A}$$

where $f_{c}$ denotes the compressive strength (MPa), F represents the failure load (N), and A is the load-bearing area (mm^2^).

## 3. Results and Discussion

### 3.1. Pore Characteristics

**Figure 3:**
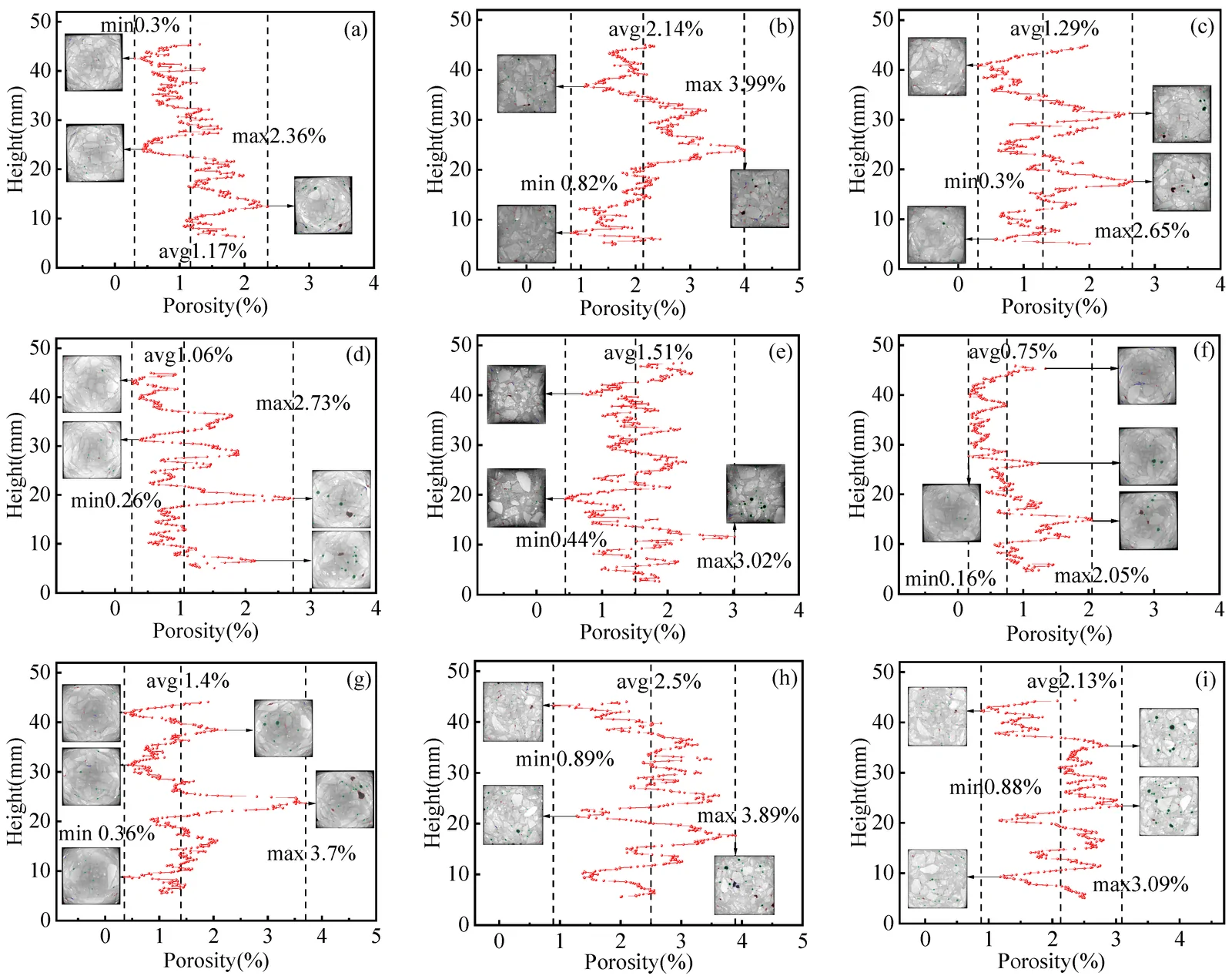
The variations in 2D slice porosity along the specimen height: (**a**) Specimen 1; (**b**) Specimen 2; (**c**) Specimen 3; (**d**) Specimen 4; (**e**) Specimen 5; (**f**) Specimen 6; (**g**) Specimen 7; (**h**) Specimen 8; (**i**) Specimen 9. Insets show representative CT slices at the indicated heights.

The variation in CT-derived slice porosity along the specimen height was evaluated using the retained axial sections. The resulting profiles describe the variation in two-dimensional pore-area fraction along specimen height. The 2D porosity distribution profiles were restricted to the central region between 5 mm and 45 mm along the specimen height to avoid the compromised pore identification accuracy typically observed at the specimen ends, where over-saturation occurs during X-ray CT scanning. The resulting 2D porosity variations along the height for all specimens are illustrated in Figure 3.

The maximum 2D porosity of the concrete specimens is predominantly concentrated in the central region, whereas the minimum porosity is observed at the extremities (Figure 3b). The observed profiles did not follow a simple monotonic top-to-bottom pattern. Casting, vibration, air escape, and curing conditions may all contribute to this spatial variation, but their separate effects were not measured. The present finding is therefore limited to the observed CT-derived two-dimensional porosity profile.

Pore sections with circularity values < 0.4 have relatively irregular two-dimensional outlines. Pore sections with circularity values from 0.4 to 0.8 exhibit intermediate two-dimensional shape regularity. This category of pores accounts for approximately 50% of the total pore population. The pore morphology approximates a perfect circle when the circularity value exceeds 0.8. Circularity was used here to compare the two-dimensional shapes of segmented pore sections. Prior work has also considered pore shape in mechanical analysis [30].

**Figure 4:**
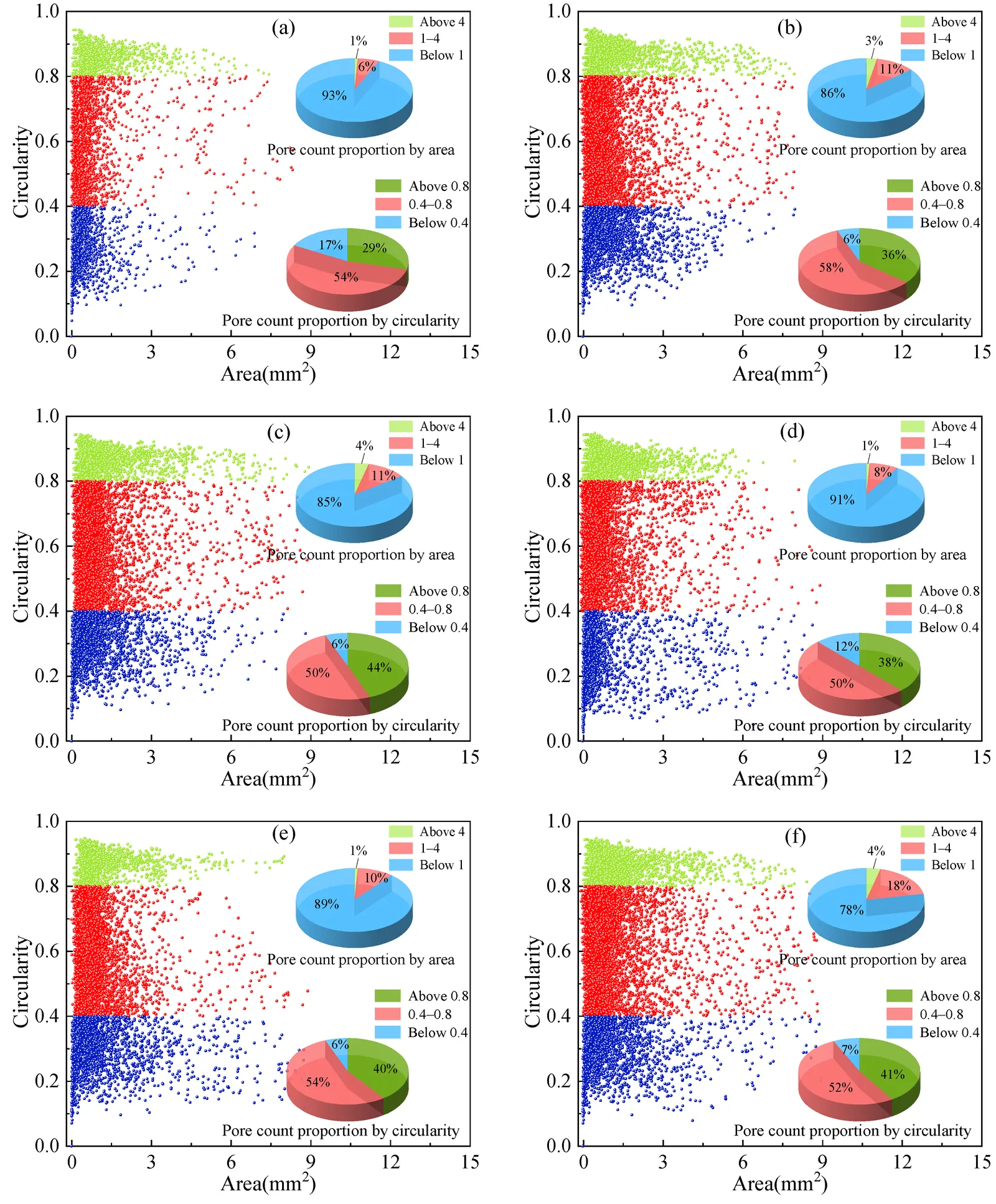

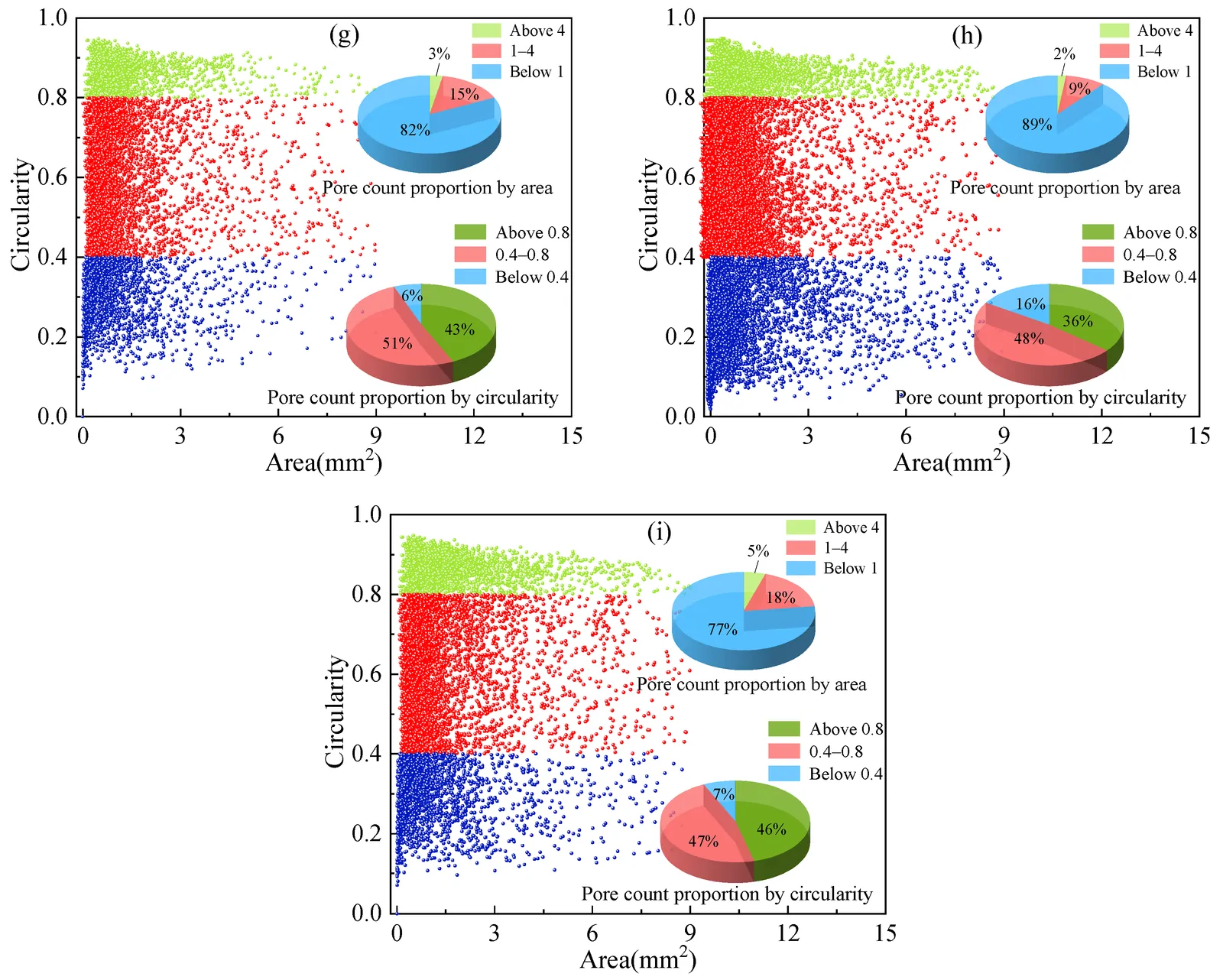
Proportion of pore counts across different pore area and circularity intervals: (**a**) Specimen 1; (**b**) Specimen 2; (**c**) Specimen 3; (**d**) Specimen 4; (**e**) Specimen 5; (**f**) Specimen 6; (**g**) Specimen 7; (**h**) Specimen 8; (**i**) Specimen 9. Scatter plots show pore-section area versus circularity, while the inset pie charts show pore-count proportions within the different area and circularity intervals.

Scatter plots were generated based on the area and circularity values of individual pores, and the pore proportion distribution across different intervals was statistically analyzed, as depicted in Figure 4. The quantity of highly circular pores (circularity > 0.8) exhibits a pronounced decreasing trend with increasing pore area across all mix proportions. By contrast, low-circularity pore sections (<0.4) showed differences in their area distributions among the mixtures, particularly in the large-area intervals.

Statistical analysis of the proportional counts reveals that pores with moderate circularity (0.4–0.8) predominate across all specimen groups. These pores consistently account for the majority (>50%), constituting the primary void structure within the concrete matrix (Figure 4a–i). The proportion of low-circularity pore sections (<0.4) varied among the mixture groups. Comparison of the area–circularity distributions can help identify mixtures with lower proportions of large-area and low-circularity pore sections.

**Figure 5:**
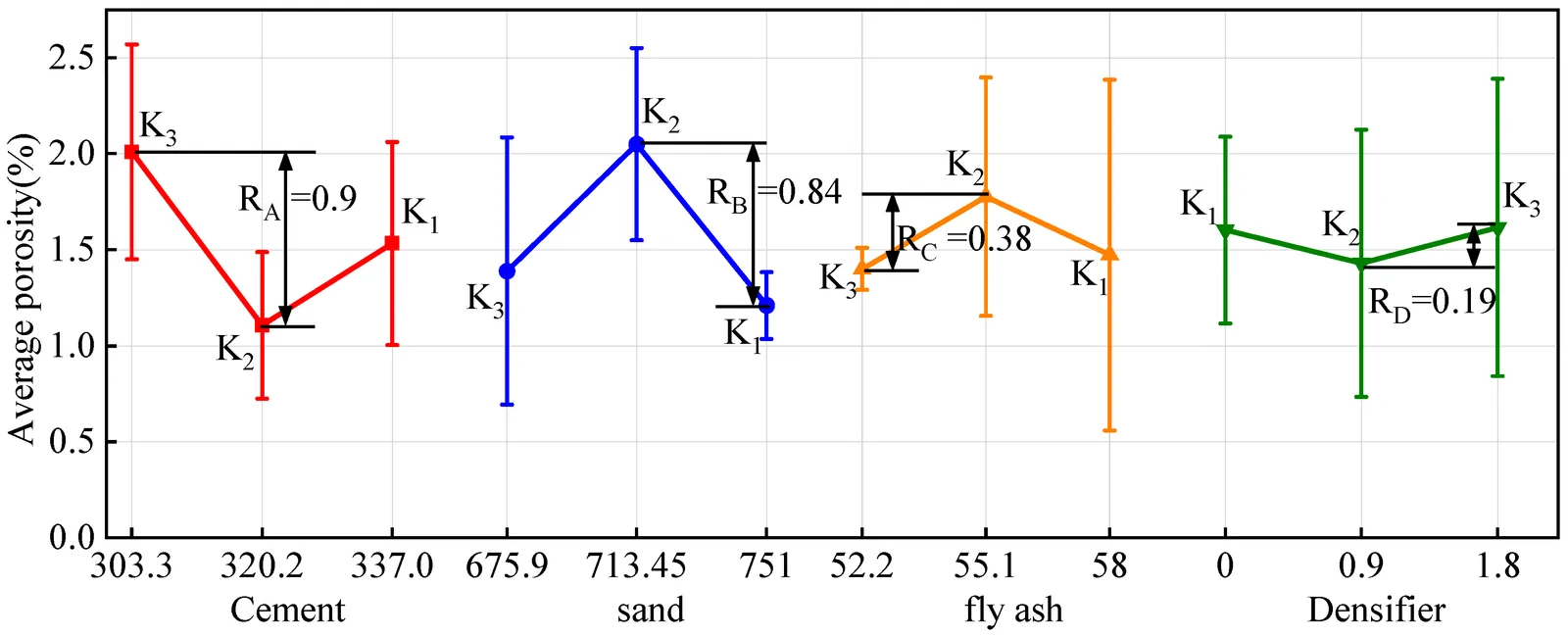
A range analysis of the average porosity.

Figure 5 presents the range analysis results for average 2D porosity, including the main-effect trends and range values (R) of each factor. The hierarchy of significance for the factors affecting porosity is A > B > C > D. As the cement content decreases, the porosity exhibits a V-shaped trend, characterized by an initial decrease followed by an increase. Conversely, reducing the sand ratio or FA content produced an inverted V-shaped main-effect trend: average slice porosity first increased and then decreased. Furthermore, an increase in the dosage triggers a similar nonlinear variation, causing the porosity to drop initially before experiencing a sharp surge. Mechanistically, cement and FA serve as the core of the cementitious system, directly governing the paste volume and the availability of hydration products. A moderate reduction in cement alongside FA may alter particle packing and the CT-resolvable pore structure. At the lowest cement level, the average slice porosity increased within the investigated mixtures. The sand ratio fundamentally modulates the void proportion between the coarse and fine aggregates. Initially, as the sand ratio decreases, the interstitial voids between the coarse aggregates enlarge. When the available paste volume is insufficient to fully occupy these voids, the porosity increases. However, upon further reduction of the sand ratio to a critical threshold, the relatively surplus paste assumes a dominant role in lubrication and void-filling, thereby driving a decrease in porosity. Overall, the cement content and sand ratio govern the paste filling and skeletal compaction processes, emerging as the most critical mix design factors governing the macroscopic average porosity. Specimen 6, coded as A_2_B_3_C_1_D_2_ according to Table 2, exhibited the lowest observed average slice porosity of 0.75%. This observed result should be distinguished from the combination derived from the individual main-effect means. The latter indicated A_2_B_1_C_3_D_2_ as the predicted combination for minimizing average slice porosity, with an additive prediction of approximately 0.50%.

The exploratory ANOVA results for the average CT-derived slice porosity are summarized in Table 3. Because the L9 array was saturated and no independent replicate CT measurements were available, factors C and D, which exhibited comparatively small sums of squares, were pooled to estimate the error term ($SS_{E}=SS_{C}+SS_{D},\;df_{E}=4$). The descriptive main-effect estimates followed the order A > B > C > D, consistent with the range analysis. Under the pooled-error assumption, the *F*-values for cement content (A) and sand ratio (B) were 8.05 and 7.71, respectively, exceeding F_0.05_(2,4) = 6.94. Conditional on pooling C and D, these *F*-values provide exploratory evidence for A and B within the investigated ranges; they should not be read as confirmatory significance tests without independent CT replication.

**Figure 6:**
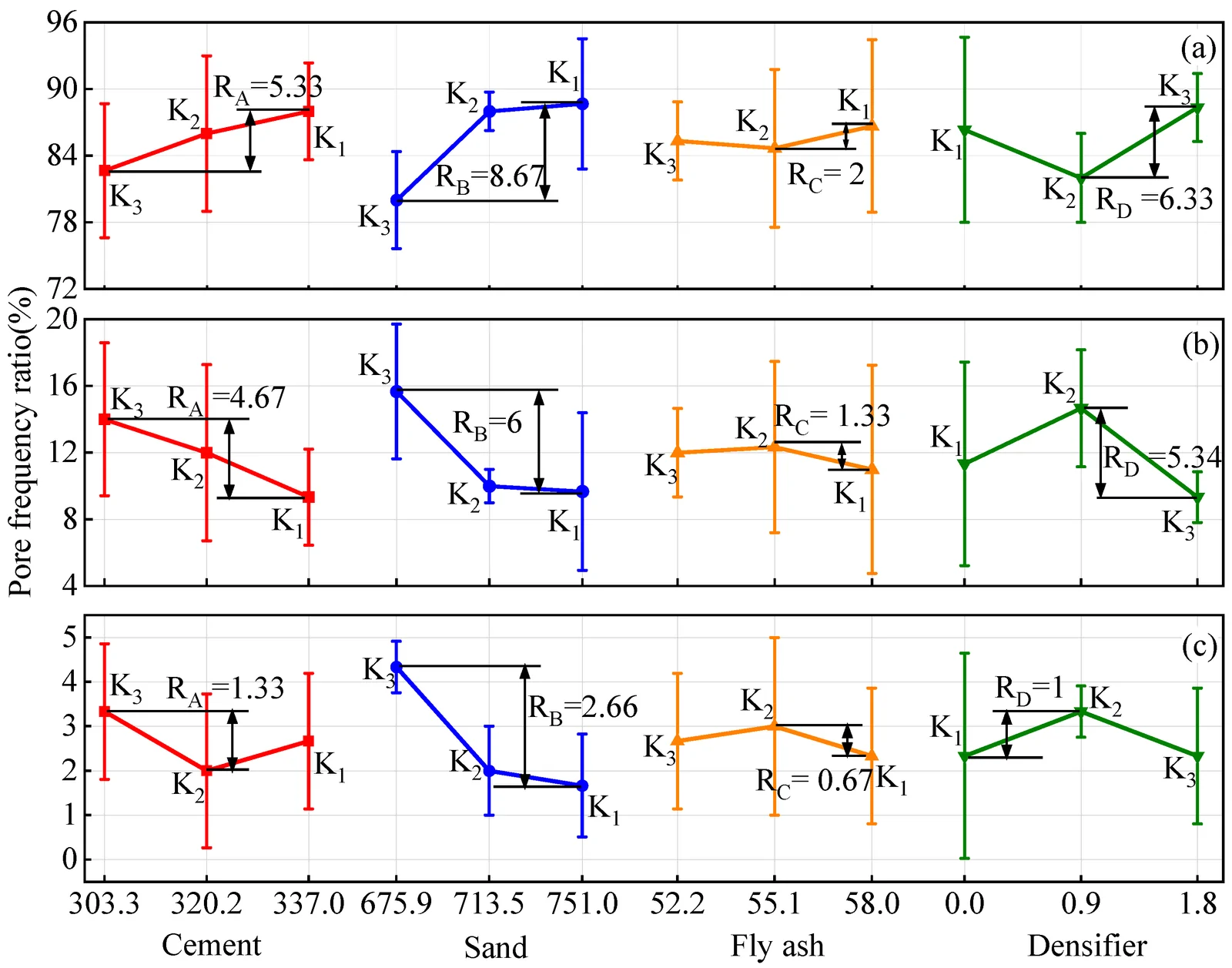
A range analysis of the proportion of pore counts across different area intervals: (**a**) <1 mm^2^; (**b**) 1–4 mm^2^; and (**c**) >4 mm^2^.

The main-effect trends of the four factors—cement content (A), sand ratio (B), FA content (C), and densification agent dosage (D)—on the proportion of pore counts across different area intervals are illustrated in Figure 6. For the area interval of <1 mm^2^ (Figure 6a), the range-value ranking is B (R = 8.67) > D (R = 6.33) > A (R = 5.33) > C (R = 2), demonstrating that the sand ratio exerts a dominant influence on the generation of minute pores. Within the 1–4 mm^2^ interval (Figure 6b), the range-value ranking follows the order B (R = 6) > D (R = 5.34) > A (R = 4.67) > C (R = 1.33). Similarly, for the >4 mm^2^ interval (Figure 6c), the order is B (R = 2.66) > A (R = 1.33) > D (R = 1) > C (R = 0.67). Across the evaluated area intervals, the sand ratio had the largest range values, indicating the strongest descriptive main-effect trend in the two-dimensional pore-section area distribution.

Fundamentally, the primary voids resulting from aggregate packing initially govern the pore structure of the concrete. The sand ratio essentially modulates the gradation and volumetric proportion between coarse and fine aggregates. According to the Packing Density Theory, an optimal sand ratio maximizes the packing density of the blended aggregates, thereby minimizing the skeletal void fraction [31]. When the sand ratio deviates from this optimal threshold (particularly upon reduction), the fine aggregates become insufficient to adequately fill the interstices between the coarse aggregates. Such packing differences are a possible explanation for variation in the observed large-area two-dimensional pore sections (>1 mm^2^). Beyond dictating the total void volume, the sand ratio critically influences the distribution of the cementitious paste by altering the total specific surface area of the aggregates. At an excessively low sand ratio, despite a reduction in the total surface area, the available cementitious paste fails to completely fill the enlarged geometric voids within the aggregate skeleton, resulting in a porous and poorly compacted matrix. Conversely, an excessively high sand ratio precipitates a dramatic increase in the total specific surface area. Under a constant paste volume, the coating layer of paste enveloping the aggregates is significantly thinned. This may contribute to differences in the measured pore-section distributions (Figure 6c). Evidently, the sand ratio governs both the total skeletal void volume and the paste distribution, playing a dominant role in determining the pore size distribution. When the 1–4 mm^2^ and >4 mm^2^ intervals were combined, the mean proportions of pore sections larger than 1 mm^2^ were 13.66%, 18.00%, and 11.66% at D_1_, D_2_, and D_3_, respectively. Thus, D_2_ produced the highest, rather than the lowest, proportion of large pore sections, whereas D_3_ produced the lowest proportion. Based on the main-effect means, A_1_B_1_C_1_D_3_ was identified as the predicted combination for minimizing the proportion of pore sections larger than 1 mm^2^. Because this combination was not included in the L9 array, it should be regarded as an unverified candidate rather than an experimentally confirmed optimum.

**Figure 7:**
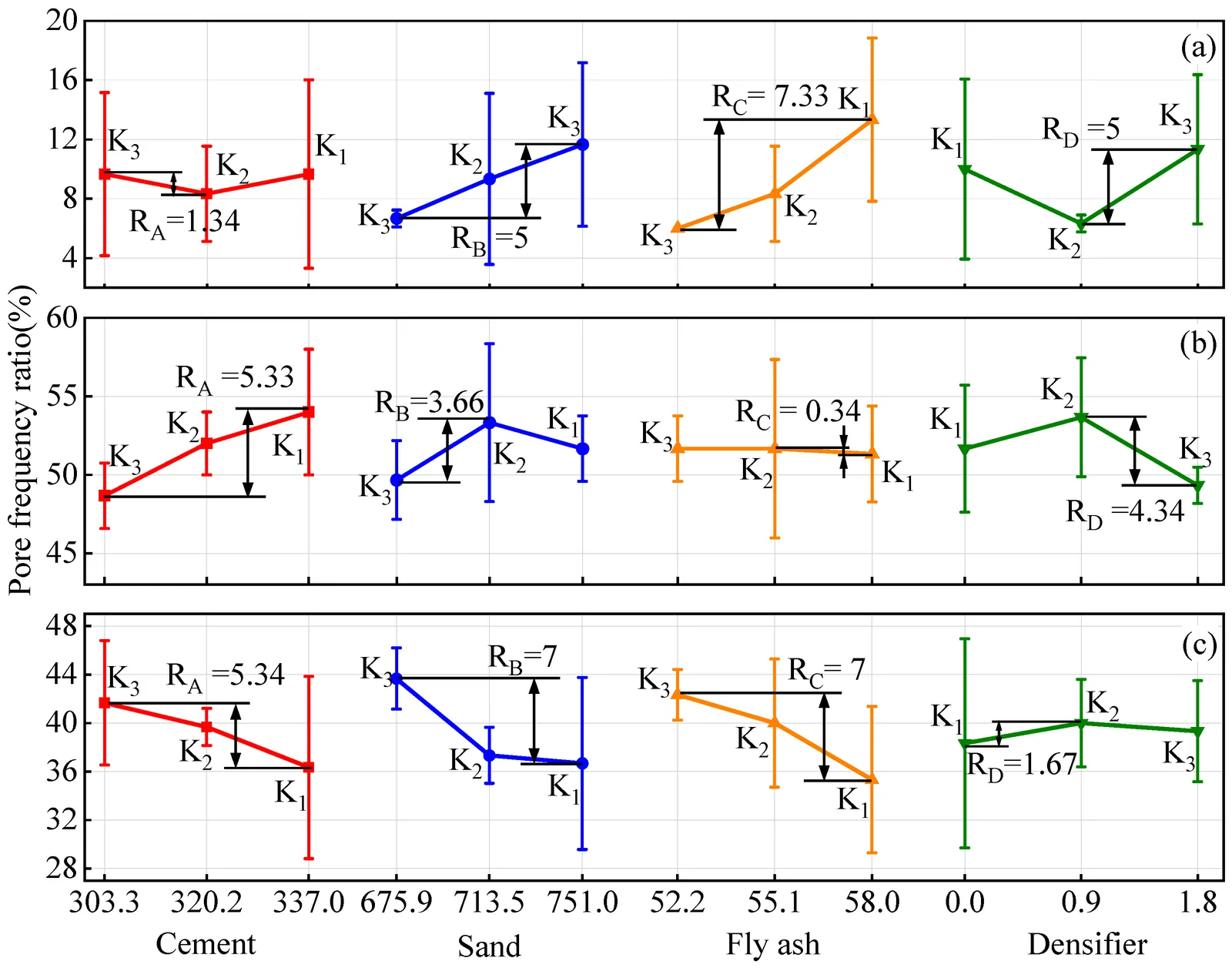
A range analysis of the proportion of pore counts across different circularity intervals: (**a**) <0.4; (**b**) 0.4–0.8; and (**c**) >0.8.

Figure 7 illustrates the influence patterns of each factor on pore circularity. As shown in Figure 7a, for circularity values < 0.4, the range-value ranking is C > B > D > A, indicating that FA had the largest range value for the low-circularity class. Within the tested mixtures, FA content was associated with differences in low-circularity pore sections. For circularity values between 0.4 and 0.8 (Figure 7b), the range-value ranking is A > D > B > C, with the cement content exerting the most prominent impact within this interval. For circularity values > 0.8, the ranking is B = C > A > D, indicating equal descriptive range values for sand ratio and FA in this class. The proportion of highly circular two-dimensional sections declined across the tested factor levels in Figure 7c. For pore sections with circularity < 0.4, the mean response values (K) were compared to identify A_2_B_3_C_3_D_2_ as a response-specific candidate combination.

The factor-level combinations predicted for the three pore-structure responses were not identical. Therefore, no single overall optimum was identified solely from the pore-structure results, and the response-specific combinations were further compared with the 28 d compressive-strength result.

### 3.2. Compressive Strength

#### 3.2.1. Evolution of Concrete Strength

**Figure 8:**
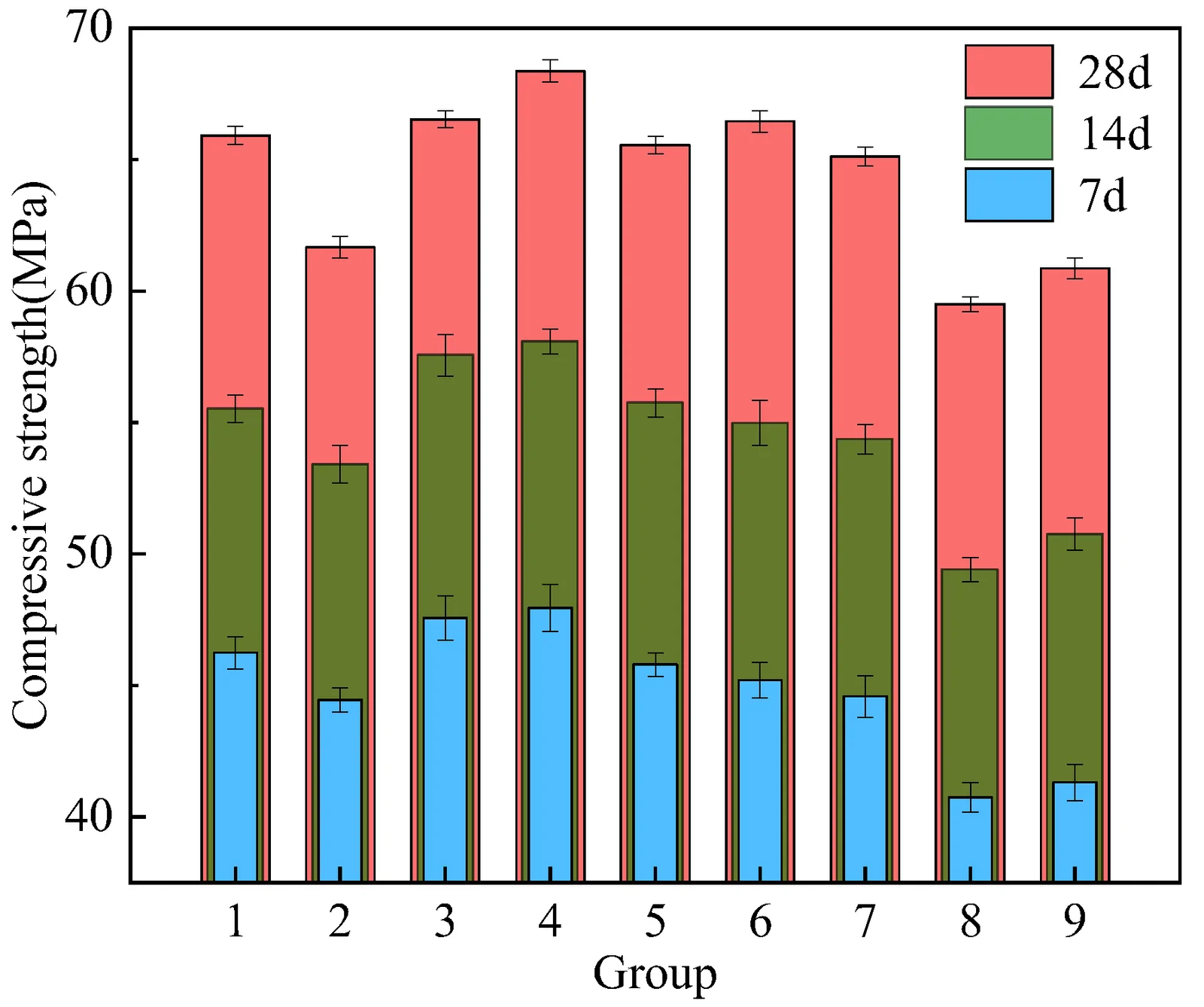
Mean compressive strength at 7, 14, and 28 d (error bars: standard deviation of three independent batches at each age).

The compressive strength of the concrete specimens was evaluated at 7, 14, and 28 d, and the corresponding results are depicted in Figure 8. Specimens 1, 3, 4, and 6 exhibited relatively high 28 d compressive strengths, with Specimen 4 achieving the maximum value of 68.37 MPa. Specimens 8 and 9 demonstrated lower 28 d compressive strengths of 59.49 MPa and 60.87 MPa, respectively.

**Figure 9:**
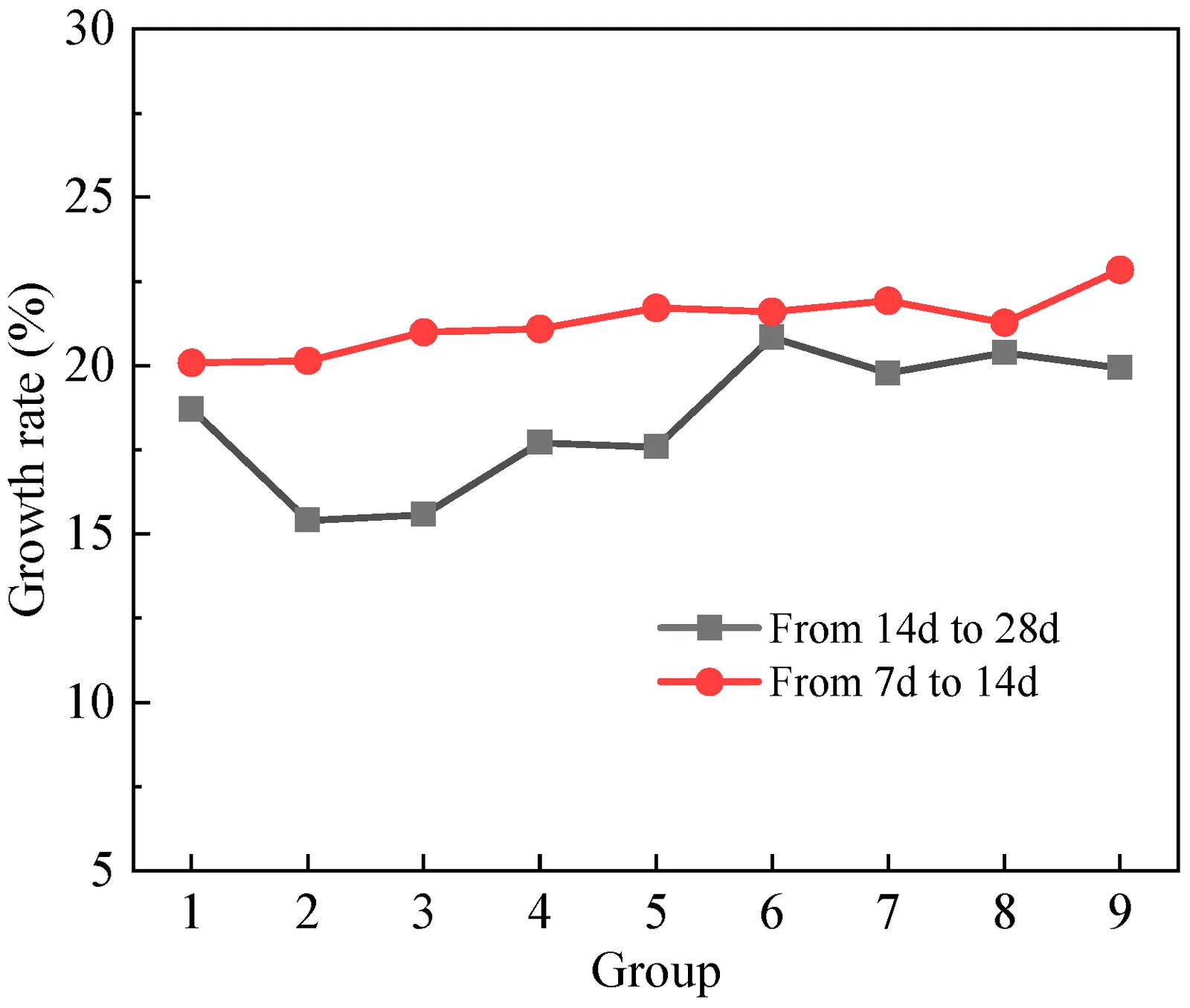
The compressive strength gain rates during the 7–14 d and 14–28 d intervals.

To further elucidate the strength development kinetics, Figure 9 illustrates that the strength growth rate from 7 to 14 d generally surpasses that from 14 to 28 d. However, for Specimens 1, 6, and 8, the strength gains during these two periods are notably comparable. Specimens 6, 7, and 9 showed comparable strength gains of approximately 20% from 14 to 28 d. Among the nine mixtures, Specimen 6 had the lowest ratio of 14 d to 28 d strength (82.75%), indicating substantial strength development over this interval.

#### 3.2.2. Range Analysis of the 28-Day Compressive Strength

**Figure 10:**
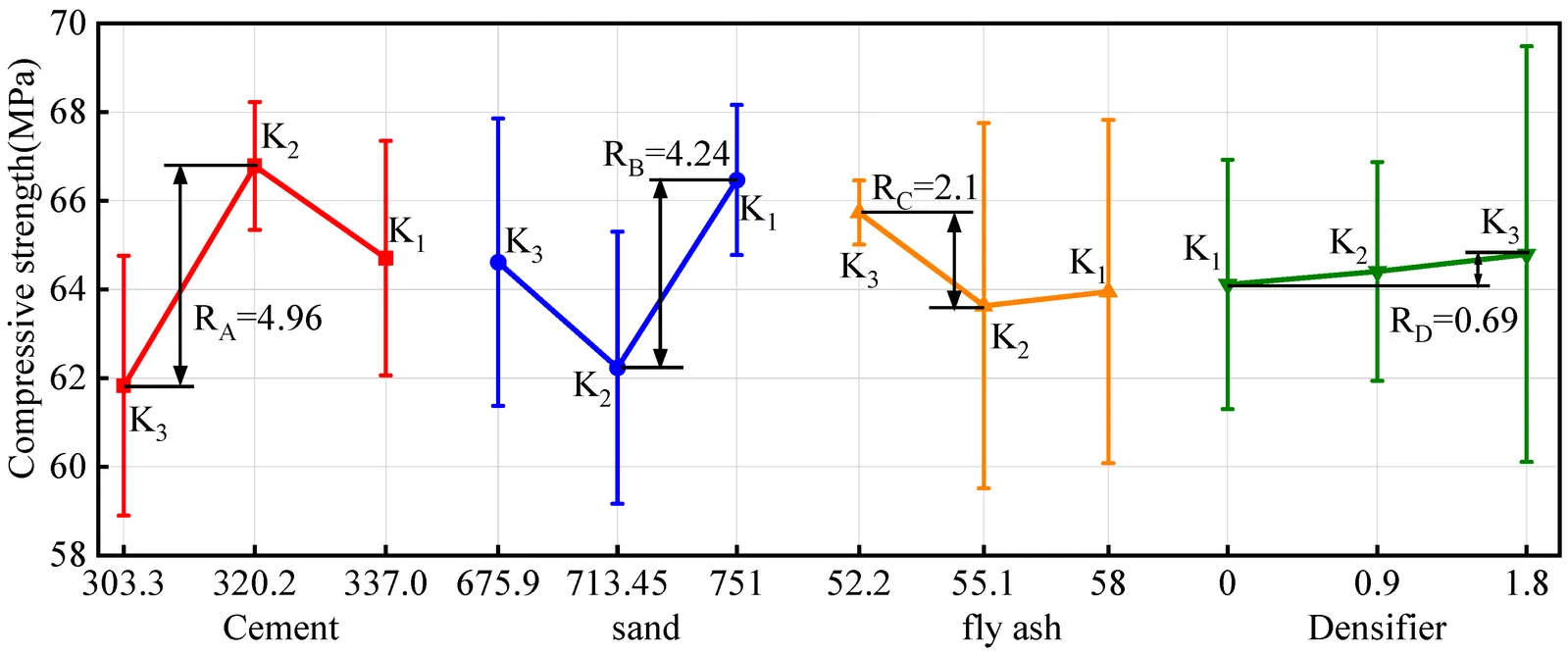
A range analysis of the 28-day compressive strength.

The main-effect trends of each orthogonal experimental factor on the 28 d compressive strength are illustrated in Figure 10. The results indicate that the range-value ranking for the factors affecting the 28 d compressive strength is A > B > C > D. Based on a comparison of the mean response values (*K*), the factor-level combination predicted to maximize 28 d strength was A_2_B_1_C_3_D_3_. Cement content emerges as the paramount factor governing 28 d compressive strength. The main-effect mean of the 28 d compressive strength was highest at the intermediate cement level A_2_ (320.15 g), reaching 66.79 MPa, compared with 64.70 MPa at A_1_ and 61.83 MPa at A_3_. The effect was therefore non-monotonic within the investigated range. Cement hydration products, particularly calcium silicate hydrate (C–S–H), contribute to the development of concrete strength.

The densifier dosage produced the smallest variation in the mean 28 d compressive strength among the four investigated factors. Its *F*-value was below the critical value in the ANOVA, indicating that the effect of densifier dosage on 28 d compressive strength was not statistically significant within the investigated range.

#### 3.2.3. Analysis of Variance (ANOVA) of the 28-Day Compressive Strength

Due to the absence of null columns in the orthogonal experimental design, the configuration represents a saturated model, where all available degrees of freedom (DOF) are allocated to the investigated factors. Consequently, no residual degrees of freedom remain to estimate the experimental error variance. For 28 d strength, three independently mixed batches per combination supplied 18 within-mixture error degrees of freedom for the reported main-effect *F*-tests.

As indicated in Table 4, the influence hierarchy of the various factors on the 28 d compressive strength is A > B > C > D, which corroborates the range analysis findings. Notably, the calculated *F*-values for cement content (A), sand ratio (B), and FA content (C) exceed the critical value F_0.01_(2,18), indicating that these three factors exert a highly significant effect on the compressive strength. In contrast, the *F*-value for the densifier dosage (D) falls below the critical threshold F_0.05_(2,18), confirming that its influence on the compressive strength is not statistically significant.

The response-specific candidate combinations were A_2_B_1_C_3_D_2_ for the average slice porosity, A_1_B_1_C_1_D_3_ for the proportion of pore sections with an area greater than 1 mm^2^, A_2_B_3_C_3_D_2_ for the proportion of pore sections with a circularity lower than 0.4, and A_2_B_1_C_3_D_3_ for the 28 d compressive strength. Levels A_2_, B_1_, and C_3_ each occurred in three of the four response-specific combinations, showing relatively consistent advantages across the evaluated responses. For Factor D, Levels D_2_ and D_3_ each occurred twice. Because the effect of densifier dosage on the 28 d compressive strength was not statistically significant, and D_2_ was associated with the lowest average slice porosity and the lowest proportion of pore sections with a circularity below 0.4, D_2_ was selected from the perspective of pore-structure refinement. For clarity, Table 5 summarizes the response-specific level selections, their criteria, and the limits of the proposed multi-response candidate.

#### 3.2.4. Relationship Between Compressive Strength and Porosity

**Figure 11:**
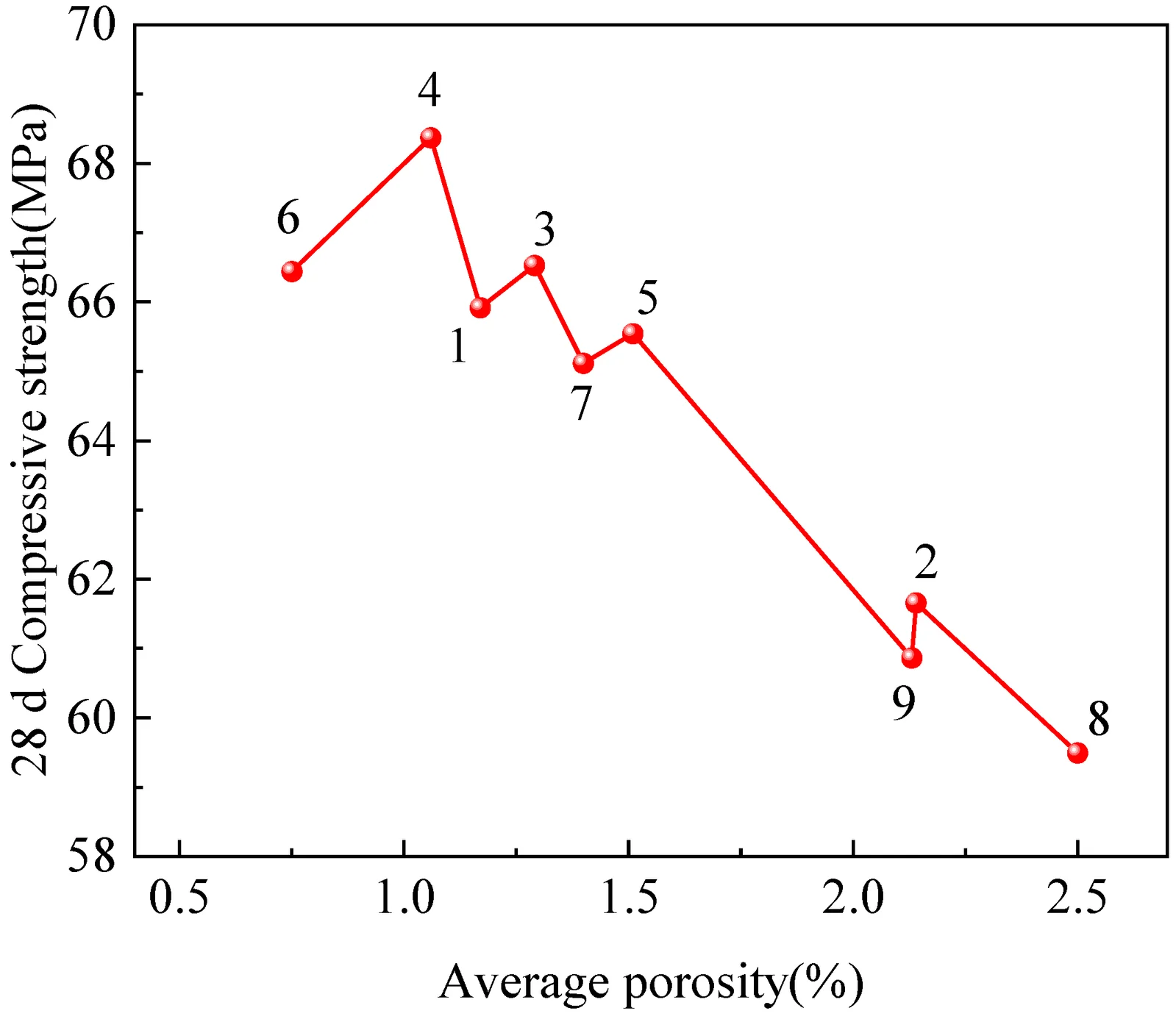
Variation in compressive strength with porosity: the numbers beside the data points indicate the specimen IDs.

The relationship between porosity and compressive strength for each specimen group was analyzed, and the corresponding correlation curve is plotted in Figure 11. The specific specimen designations are denoted by numerical labels adjacent to the data points. Generally, the compressive strength exhibits a progressive decline as porosity increases. However, despite possessing the minimal overall porosity (mean porosity of 0.75%), the compressive strength of Specimen 6 ranks only third among the nine experimental groups. This difference was considered alongside its pore-size distribution. Although the total porosity is low, the proportion of large-sized pores (area > 1 mm^2^) in Specimen 6 reaches 22%, compared to 9% in Specimen 4 and 15% in Specimen 3. This value of 22% is second only to that of Specimen 9 (23%), whereas all remaining specimens—except Specimen 7 (18%)—exhibit large-pore proportions below 15%. Notably, both Specimens 7 and 9 had lower compressive strengths than some specimens with higher mean porosity, indicating that average porosity alone did not fully describe the strength ranking. Overall, compressive strength was negatively associated with average slice porosity, while pore-size distributions differed among the tested mixtures.

Statistical analysis corroborates that the cement content and sand ratio are the primary factors governing the 28 d compressive strength of the concrete, whereas the densifier dosage showed no statistically significant effect on the 28 d compressive strength within the investigated range. The CT-derived pore-section area and circularity distributions also differed among the tested mixtures, complementing the average porosity comparison.

### 3.3. Nonlinear Regression Analysis

To investigate the observed relationship between average slice porosity and 28 d compressive strength, a univariate nonlinear regression analysis based on the ordinary least squares method was employed. A natural logarithm (ln) transformation was applied to the independent variable (average porosity, X) during the model construction, and a second-order polynomial in ln(X) was adopted to describe curvature in the observed porosity–strength data.

The fundamental expression of the finalized regression model is formulated as follows:

(13) $$Y=\beta_{0}+\beta_{1}\ln\left(X\right)+\beta_{2}{\left[\ln\left(X\right)\right]}^{2}+\varepsilon$$

where Y denotes the compressive strength of the specimens (MPa); X represents the average porosity (%); $\beta_{0}$ is the model intercept, corresponding to the fitted compressive strength at 1% average slice porosity (X = 1); $\beta_{1}$ is the first-order regression coefficient with ln(X), describing the first-order contribution of ln(X) to the empirical fit; $\beta_{2}$ is the second-order regression coefficient associated with [ln(X)]^2^, describing the curvature of the fitted porosity–strength relationship; and ε stands for the random error term.

**Figure 12:**
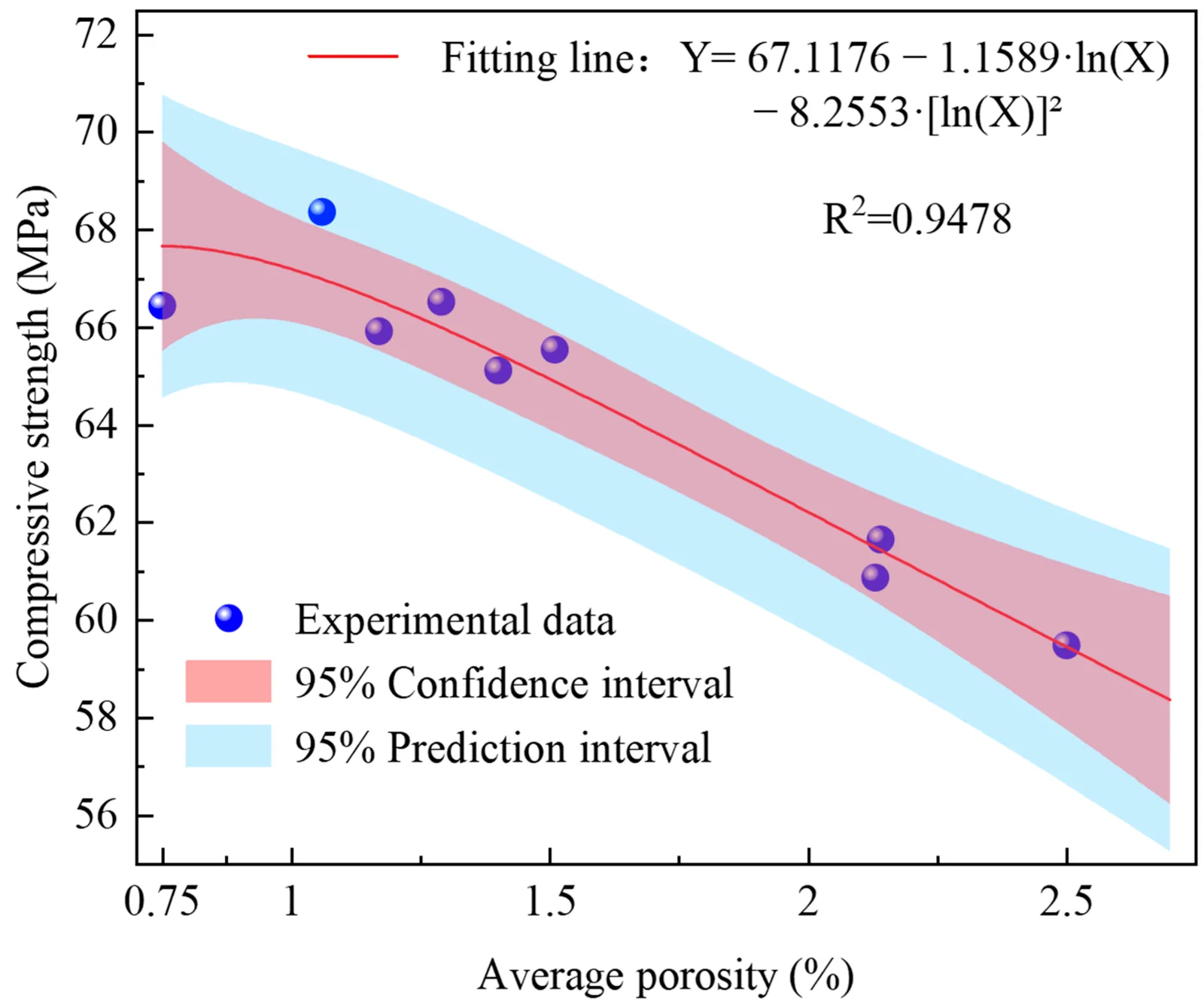
Logarithmic regression analysis between compressive strength and average porosity.

For X, expressed as average slice porosity in percent form, the fitted equation is Y = 67.1176 − 1.1589 ln(X) − 8.2553[ln(X)]^2^; the coefficient estimates and their 95% confidence intervals are presented in Table 6. With average slice porosity expressed as a percentage value, the intercept of 67.1176 MPa represents the fitted 28 d compressive strength at a porosity of 1%. The coefficient of the squared logarithmic term describes the curvature of the fitted relationship. The resulting curve is shown in Figure 12.

The overall regression results are summarized in Table 7. For the nine investigated specimen groups, the second-order logarithmic model achieved an R^2^ of 0.948 and an adjusted R^2^ of 0.930. The overall *F*-statistic was 54.46 (df = 2, 6; *p* < 0.001), indicating a close fit to the observed data. As illustrated in Figure 12, the fitted relationship has a shallow maximum near 0.93% porosity and shows an overall decline in compressive strength as porosity increases across the remaining observed range.

To assess the stability of the fitted relationship, each of the nine specimen groups was omitted in turn. The models were fitted using the remaining eight groups and then used to estimate the 28 d compressive strength of the omitted group. Based on these nine held-out estimates, the RMSE was 1.422 MPa for the second-order logarithmic model and 1.317 MPa for the linear porosity model. The similar held-out errors do not establish a predictive advantage for the more complex curve, which is retained as an empirical description of the nine tested mixtures. The residuals of the second-order logarithmic fit ranged from −0.812 to 1.349 MPa. Influence analysis indicated that Specimen 6 contributed substantially to the curvature near the low-porosity end of the fitted curve (leverage = 0.906; Cook’s distance = 5.62).

## 4. Conclusions

Based on X-CT analysis and compressive-strength tests of the nine concrete mixtures, the following conclusions were drawn:

The CT-resolvable two-dimensional pore-section morphology and size distribution varied among the investigated mix designs. Cement content and sand ratio showed the largest main-effect trends for average slice porosity. The FA content was associated with changes in the pore-section area and circularity distributions. Within the investigated range, higher FA contents were associated with a higher proportion of pore sections with a circularity lower than 0.4.

Across the nine tested mixtures, 28 d compressive strength was negatively associated with CT-derived average slice porosity. The cement content and sand ratio are the paramount factors governing the baseline compressive strength grade. FA content also showed a significant main-effect trend in 28 d compressive strength within the investigated range. Within the investigated dosage range of 0–1.8 g per laboratory batch, the 28 d strength data did not provide evidence of a densifier main effect at the 5% significance level (*p* = 0.088).

The second-order logarithmic regression model described the observed association between average 2D porosity and 28 d compressive strength across the nine investigated specimen groups (R^2^ = 0.948). Leave-one-group-out cross-validation yielded an RMSE of 1.422 MPa, comparable to 1.317 MPa for a linear porosity model. These results support the use of X-CT-derived porosity as an empirical indicator of strength variation within the investigated range; additional specimen groups are needed to assess the model’s applicability to other mix designs.

Comparison of the four response-specific candidate combinations showed that Levels A_2_, B_1_, and C_3_ each appeared in three of the four combinations. For Factor D, Levels D_2_ and D_3_ each appeared twice. Considering that the densifier dosage had no statistically significant effect on the 28 d compressive strength and that D_2_ was favored by two pore-structure responses, A_2_B_1_C_3_D_2_ was provisionally proposed as an exploratory, consensus-based candidate mixture for balancing pore-structure characteristics and compressive strength.

A limitation of the present study is that the proposed A_2_B_1_C_3_D_2_ combination was inferred from the response-specific main-effect trends and was not directly included in the L9 orthogonal array. Therefore, its comprehensive performance has not yet been experimentally confirmed. In addition, the present design primarily evaluated the main effects of the four investigated factors and did not independently quantify possible interactions among them. Future work should conduct independent confirmation tests on the proposed combination using separately prepared batches and corresponding X-CT and compressive-strength measurements. The reported CT descriptors are conditional on the stated histogram equalization, threshold, and morphological closing settings; threshold/filter sensitivity and manual-segmentation agreement were not independently tested.

## Figures and Tables

**Table 1:** Experimental materials and physicochemical properties.

Material Designation	Type	Property	Value
Cement	P.O42.5	Quality status	Qualified
Sand	River sand	Fineness modulus	2.7
		Silt content (%)	2.5
Coarse aggregate	Crushed stone	Grading (mm)	5–15
		Silt content (%)	0.8
Water	Tap water	/	/
FA	Class F, Grade II	Fineness (45 μm sieve residue, %)	≤30.0
GGBS	S95 grade	Specific surface area (m^2^/kg)	≥400
Superplasticizer	XYL-1 (High-performance)	Water reduction rate (%)	30%
		Chloride ion content (%)	≤0.02%
Densifier	MATE-ADV	Density (g/cm^3^)	1.02 ± 0.02
		Solid content (%)	≥7.20, <8.80

**Table 2:** Orthogonal experimental design matrix for concrete mix proportions.

No.	A: Cement (g)	B: Sand Ratio (%)	Sand Mass (g)	C: FA (g)	D: Densifier (g)	Coarse Aggregate (g)	GGBS (g)	Superplasticizer (g)	W/B
1	337	43.51	751.0	58	0	975	54	11.2	0.37
2	337	42.25	713.45	55.1	0.9	975	54	11.2	0.37
3	337	40.94	675.9	52.2	1.8	975	54	11.2	0.37
4	320.15	43.51	751.0	55.1	1.8	975	54	11.2	0.37
5	320.15	42.25	713.45	52.2	0	975	54	11.2	0.37
6	320.15	40.94	675.9	58	0.9	975	54	11.2	0.37
7	303.3	43.51	751.0	52.2	0.9	975	54	11.2	0.37
8	303.3	42.25	713.45	58	1.8	975	54	11.2	0.37
9	303.3	40.94	675.9	55.1	0	975	54	11.2	0.37

**Table 3:** The analysis of variance (ANOVA) results for the average porosity.

Factors	Degree of Freedom	Sum of Squares	Mean Square	F	Critical Value	Significance
A	2	1.2253	0.6126	8.052	F_0.05_(2,4) = 6.94	*
B	2	1.1736	0.5868	7.713	F_0.05_(2,4) = 6.94	*
Pooled Error C + D	4	0.3043	0.0761			
Total	8	2.7032				

Note: Because factors C and D exhibited comparatively small sums of squares within the investigated factor ranges, their sums of squares and degrees of freedom were pooled to provide an estimate of experimental error ($SS_{E}=SS_{C}+SS_{D},\;df_{E}=df_{C}+df_{D}=4$). Pooling assumes that the C and D contributions to this response are negligible. If either factor has a genuine effect, the pooled mean square is not a pure error estimate. Without independent CT replicates, no assumption-free error estimate is available to confirm the nominal F-test results. The asterisk (*) indicates statistical significance at the 0.05 level based on the pooled-error F-test.

**Table 4:** The ANOVA results for the 28-day compressive strength.

Factors	Degree of Freedom	Sum of Squares	Mean Square	F	Critical Value	Significance
A	2	111.51	55.76	145.46	F_0.01_(2,18) = 6.01	*
B	2	81.32	40.66	106.07	F_0.01_(2,18) = 6.01	*
C	2	22.99	11.5	29.99	F_0.01_(2,18) = 6.01	*
D	2	2.13	1.07	2.79	F_0.05_(2,18) = 3.55	/
Error	18	6.9	0.3833			
Total	26	224.85				

Note: The asterisk (*) indicates statistical significance at the 0.01 level (*p* < 0.01).

**Table 5:** Response-specific main-effect candidates and selection basis.

Response	Levels	Selection Criterion	Limitation
Average slice porosity	A_2_B_1_C_3_D_2_	Lowest main-effect estimate	Untested combination
Area > 1 mm^2^ sections	A_1_B_1_C_1_D_3_	Lowest main-effect estimate	Untested combination
Circularity < 0.4 sections	A_2_B_3_C_3_D_2_	Lowest main-effect estimate	Untested combination
28 d compressive strength	A_2_B_1_C_3_D_3_	Highest main-effect estimate	Untested combination
Multi-response candidate	A_2_B_1_C_3_D_2_	A_2_/B_1_/C_3_ frequency; D_2_ favored by two pore responses	Exploratory; confirmation required

**Table 6:** Coefficients table.

	Coef	Std Err	t	95%CI [0.025, 0.975]
Const	67.1176	0.3806	176.33	[66.186, 68.049]
X_1_ (lnX)	−1.159	1.519	−0.763	[−4.875, 2.557]
X_2_ [ln(X)]^2^	−8.255	1.909	−4.324	[−12.926, −3.584]

**Table 7:** OLS regression results.

Model	Observations	df Residuals	R^2^	Adjusted R^2^	F	*p*
Ordinary Least Squares	9	6	0.948	0.930	54.46	0.000142

## Data Availability

The original contributions presented in this study are included in the article. Further inquiries can be directed to the corresponding author.
